# Impact of SWMM Fouling and Position on the Performance of SWRO Systems in Operating Conditions of Minimum SEC

**DOI:** 10.3390/membranes13070676

**Published:** 2023-07-18

**Authors:** Alejandro Ruiz-García, Mudhar A. Al-Obaidi, Ignacio Nuez, Iqbal M. Mujtaba

**Affiliations:** 1 Department of Electronic Engineering and Automation, University of Las Palmas de Gran Canaria, Campus Universitario de Tafira, 35017 Las Palmas de Gran Canaria, Spain; ignacio.nuez@ulpgc.es; 2 Department of Computer Techniques, Technical Institute of Baquba, Middle Technical University, Baquba 00964, Iraq; dr.mudhar.alaubedy@mtu.edu.iq; 3 Department of Chemical Engineering, Faculty of Engineering and Informatics, University of Bradford, Bradford BD7 1DP, UK; i.m.mujtaba@bradford.ac.uk

**Keywords:** desalination, reverse osmosis, membranes, fouling, energy consumption, simulation

## Abstract

Due to water stress in the world in general desalination technologies are becoming increasingly important. Among the available technologies, reverse osmosis (RO) is the most widespread due to its reliability and efficiency compared to other technologies. The main weakness of RO is the loss of performance due to membrane fouling, which usually affects the water permeability coefficient (*A*), causing it to decrease. In RO desalination plants, fouling does not affect all spiral wound membrane modules (SWMMs) in the pressure vessels (PVs) in the same way. This will depend on the type of fouling and the position of the SWMM inside the PV. In this study, the impact of *A* and the position of the SWMM on the performance of the RO system is analyzed. For this purpose, decrements of up to 50% have been assumed for the seven SWMMs in series considering nine commercial SWMM models. The operating point analyzed is that which minimizes the specific energy consumption (*SEC*), a point obtained in a previous work carried out by the authors. The results show how the impact of *A* on the SWMM in the first position is more significant than the impact on modules that are in another position for the nine SWRO models studied. A drop of 50% in the coefficient *A* of the first element produces a permeate loss in the pressure pipe between 0.67 and 1.35 m${}^{3}$ d${}^{-1}$. Furthermore, it was observed that the models with the lowest coefficient *A* exhibited the highest performance losses in terms of permeate production when *A* was decreased.

## 1. Introduction

The shortage of water suitable for human consumption and other activities has been at least partially remedied by desalination for several decades [1]. Among the technologies available on a large scale to desalinate both seawater and brackish water [2], the most widespread is reverse osmosis (RO) [3,4]. This is due to its better energy efficiency compared to other technologies [5,6]. Climate change causes a rise in global temperatures, which leads to increased water stress [7]. This will drive the installation of desalination plants where they were not necessary just a few years ago. New technologies continue to be studied to efficiently desalinate seawater and brackish water. These include forward osmosis [8,9], pressure-retarded osmosis [10,11], osmotic distillation [12] and thermo-osmosis [13]. However, RO remains the most widespread technology [14]. Although the RO process is more efficient than others from the perspective of energy consumption, it is still a relatively intensive process regarding the energy required due to certain limitations [15]. It is crucial to investigate how to make this process more efficient through the development of, for example, more efficient and fouling-resistant membranes [16,17], improved energy recovery devices (ERDs) [18,19], enhanced automation and control [20], optimal RO system design [21] and the determination of optimal operating points to reduce the energy required [22,23,24].

### 1.1. Optimization of RO Systems

Many studies have been conducted in relation to the optimization of RO systems and the impact of fouling on their efficiency [25,26,27]. Regarding the optimization of RO system design and operation, Lu et al. [28] explored the optimal design of seawater (SWRO) and brackish water (BWRO) systems using different spiral wound membrane modules (SWMMs) and considered the use of ERDs and different stages to minimize total annualized costs. Vince et al. [29] carried out a multi-objective optimization of BWRO desalination plants, while Li [30,31] used constrained nonlinear optimization to minimize specific energy consumption (*SEC*) in RO systems. Du et al. [32] proposed an optimization method for BWRO and SWRO desalination plants with SWMMs and later [33,34] carried out further studies on optimization methods considering different RO system designs. Li and Noh [35] validated a model used for optimization of the operation of a BWRO desalination plant. Jiang et al. [36] studied the optimal operation of a full-scale SWRO desalination plant with four storage tanks, while Du et al. [37] explored the optimization of SWRO systems for boron removal. The studies considered different factors, including feedwater concentration, membrane modules, stages, ERDs, inter-stage pumps, and water recovery rates. Constraints, such as permeate quality, were also taken into account.

Kotb et al. [38] conducted an intriguing research study that evaluated the optimization of SWRO process configurations and operating conditions. They assessed systems with various stages and retentate bypass. They also took into consideration ERDs and finally recommended that permeate flows of between 144 m${}^{3}$ d${}^{-1}$ and 288 m${}^{3}$ d${}^{-1}$ were suitable for RO systems with one and two stages, respectively, to minimize production costs. Ahunbay et al. [39] investigated the minimization of the *SEC* of SWRO systems using a multi-stage arrangement comprising nanofiltration SWMMs, achieving flow recovery (*R*) rates of about 65%. Alsarayreh et al. [40] aimed to minimize and assess the *SEC* of an existing BWRO desalination plant by changing the operating conditions and incorporating an ERD, obtaining reductions in the range of 47–53.8% compared to the original design without the ERD. Kim et al. [41] optimized SWRO systems with two stages and SW400R SWMMs from LG Chem, considering ERDs and focusing on energy efficiency. Chu et al. [42] conducted a study to determine the optimal design and operating conditions of a full-scale SWRO desalination plant with two trains, both with ERD and a second pass with BWRO SWMMs. In both studies, they used the manufacturer’s software (CSMPRO5, https://www.csmfilter.com/csm/03result/Software.asp, Toray, Tokyo, Japan) to evaluate the installation of 16-inch SWMMs from Toray and concluded that the optimal energy-efficient split partial ratio was 4.5:5.5, satisfying a final permeate concentration ($C_{p}$) requirement of 0.3 g L${}^{-1}$ or less and a normalized *SEC* of less than 0.1 kWh m${}^{-3}$. The objective of another study by the same group [43] was to identify operational strategies that could lower the costs associated with running and maintaining BWRO desalination plants in South Korea. To this end, they examined the potential benefits of incorporating low pressure SWMMs and attained to a reduction in *SEC* of approximately 16%.

### 1.2. Fouling Impact on RO System Performance

Membrane fouling is perhaps the weakest point in RO processes [16,17]. Fouling can be organic [44,45], inorganic [46,47] and biological [48,49]. In terms of performance, fouling mainly has two types of impact. Firstly, it decreases the water permeability coefficient (*A*) of the membrane, which implies having to increase the feed pressure to maintain production, which translates in turn into an increase in *SEC* [50,51]. Secondly, it causes an increase in the solute permeability coefficient (*B*), which leads to an increase in the passage of salts through the membrane, which in turn can compromise the use of the permeate due to a worsening of its quality in terms of concentration [52]. An additional consequence is the increase in pressure drop across the membrane elements due to the deposition of fouling agents on the membrane surface [52]. As the pressure drop increases, the efficiency of the process in terms of specific energy consumption also decreases.

The fouling impact on permeate flux ($J_{p}$) and therefore on *A* in long-term operation was studied by Wilf et al. [53]. The experimental data of three years of operation from different SWRO desalination plants were used. They observed decrements between 25 and 20% in the $J_{p}$ of the entire RO system. Four years of operating data from a full-scale SWRO desalination plant were used by Mohamed et al. [54] to study performance decline due to fouling effects. The installed SWMM was the TFC 2822 Fluid system™, the initial feed pressure ($p_{f}$) was 67 bar and the flux recovery *R* ranged between 26–33%. A 44% reduction in terms of $J_{p}$ was observed. Abbas et al. [55] studied the performance decline of the SWMM BW30-400 Filmtec™in a full-scale BWRO desalination plant with data obtained from five years of operation. It was found that over an operating period of 500 days, the *A* for the overall plant decreased by about 25% and salt rejection dropped by 1.9%. Belkacem et al. [56] observed a performance decline of the SWMM BW30LE-440 Filmtec™in a BWRO desalination plant with two stages and with re-circulation. A 10% performance decline in 20 weeks of operation in terms of $J_{p}$ was seen. Fouling results in changes to the operating conditions in RO desalination plants to maintain permeate production, modifying the optimal operating points. Sassi and Mujtaba [57] optimized an existing BWRO system with three stages taking into consideration the effect of fouling on performance in a simulation-based study. They determined a 20% in savings in terms of *SEC* compared to the base case. Park et al. [58] carried out a simulation-based study of a full-scale SWRO system for boron removal considering fouling. The fouling effect was applied through the mass transfer coefficient (*k*) as fouling enhances the concentration polarization, and so the resistance increase due to fouling was ignored. An increment of 0.78 kWh m${}^{-3}$ was obtained due to the fouling effect. A cost optimization study on the BWRO process considering fouling was done by Ang et al. [59]. Nanofiltration and BWRO SWWMs were considered in a quite short operating time during a laboratory-scale experiment. $J_{p}$ decreased at an average 5% for all the membranes, which was attributed to fouling phenomena. Kim and Hong [60] optimized SWRO systems with an internally staged design method. A total of 36 combinations with three commercial SWMMs from LG Chem and pressure vessels (PVs) with 7 SWMMs in series were assessed. They found that the internally staged combinations were more effective in terms of *SEC* when the SWMMs were fouled under high *R* and flux conditions.

In RO systems, the SWMMs are arranged in series in PVs, and fouling does not affect all modules equally [61,62,63]. Usually, the fouling effect is measured for the entire RO system due to the distribution of available sensors, which are usually at the beginning and end of each stage. This does not allow for an evaluatation of the impact of fouling inside the PV, and therefore also which SWMM is most affected by the fouling and is more susceptible to being replaced than others so that the performance of the RO desalination plant is not so affected.

The aim of this study is to evaluate the performance of an SWRO system with seven SWMMs in series and nine different models of commercial SWMMs, considering *A* reductions of individual elements as an estimate of the effect of fouling on these elements.

## 2. Materials and Methods

### 2.1. Commercial SWMMs

The characteristics of commercial SWRO SWMMs employed in this study are given in Table 1. The SWMMs used as well as the main characteristics in terms of membrane surface (*S*${}_{m}$), feed channel height (*h*) and water and solute permeability coefficients *A*${}_{0}$ and *B* are shown in Table 1. In order to make the results obtained in the simulations more realistic, we considered certain restrictions for different SWMMs that are usually recommended by the membrane manufacturers in terms of maximum feed flow ($Q_{f}$) (384 m${}^{3}$ d${}^{-1}$), maximum $Q_{p}$ (33.36 and 36.24 m${}^{3}$ d${}^{-1}$ for the SWMMs with *S*${}_{m}$ of 37.16 and 40.88 m${}^{2}$, respectively), minimum brine flow ($Q_{b}$) (72 m${}^{3}$ d${}^{-1}$) and maximum *R* (16%). For the nine SWMMs considered, the same porosity in the feed-brine channel (ε) (0.89), friction factor (λ) and Sherwood number (*Sh*) were estimated, as characteristics of feed spacer geometries of the SWMMs are not supplied by the membrane manufacturers.

### 2.2. Equations and Simulation Algorithm for SWRO Systems

**Figure 1:**
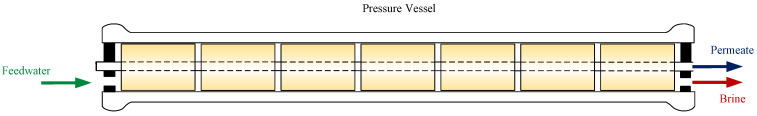
Flow diagram of the PV with 7 SWMMs in series.

The equations used to estimate the SWRO system performance came from the solution–diffusion transport model [1,65], which is commonly used for simulating RO systems as it usually provides results close to the real behaviour of this process. The equations were applied considering averages per SWMM and in a sequential manner, with the outputs of the first SWMM used as the inputs of the second SWMM. For this study, only 1 PV with 7 SWMMs in series was considered (Figure 1), since for SWRO desalination plants with higher production capacity the results obtained would simply have to be multiplied by the number of PVs desired in the SWRO desalination plant. The variation of temperature *T* and pressure drop in the permeate side along the SWMMs and PV were disregarded. The algorithm used for the performance estimation of the SWRO system can be found in a previous study [21]. To determine all the above variables, the aforementioned algorithm was implemented in MATLAB^®^ 2021b. The operating point that minimizes the energy consumption for each of the 9 SWMMs studied was considered for 7 SWMMS in series and $C_{f}$ = 30 g L${}^{-1}$. These operating points were obtained in a previous study [64]. This means that $p_{f}$ and $Q_{f}$ are already established for each SWMM model, as shown in Table 2. In this study, decreases in the *A* coefficient from 10% to 50% were considered for each of the SWMMs in series and for each of the 9 SWMM models considered. That is, it was assumed that the first SWMM of the 7 in series suffers fouling such that it loses performance and the *A* coefficient is reduced while the rest of the SWMMs in series continue with the initial $A_{0}$ coefficient (new SWMM). Subsequently, the same approach is used with the second SWMM considering that the other 6 SWMMs have not been fouled, and so on up to the seventh element. This allows estimating the impact of SWMM fouling on the SWRO system performance even if the SWMMs are exchanged in position once they are affected by fouling in terms of reduction of the *A* coefficient. For this purpose, the transport equations shown in Table 3 were used. This study did not consider variations in coefficient *B* due to fouling. It should be noted that fouling can also cause the pressure drop to increase along the PV [66], which was also not considered in this study.

The performance loss due to fouling was quantified through the flow factor ($FF_{i}$) which multiplies the coefficient $A_{0}$ of the SWMM in the position *i* ($A_{i}$). The temperature correction factor (*TCF*${}_{i}$) was assumed to be 1 ($T_{{\mathrm{fb}}_{i}}$ = 25 ${}^{\circ }$C for all SWMMs). *TMP*${}_{i}$ is the transmembrane pressure, $\Delta p_{i}$ is the pressure gradient across the membrane, $\Delta \pi_{i}$ is the osmotic pressure gradient across the membrane, $p_{p_{i}}$ is the permeate pressure (34,473.8 Pa for all SWMMs), $\pi_{m_{i}}$ is the osmotic pressure on the membrane surface, $\pi_{p_{i}}$ is the osmotic pressure in the permeate, $L_{i}$ is the SWMM length (1 m for all SWMM models), $v_{{\mathrm{fb}}_{i}}$ is the feed-brine velocity, $\lambda_{i}$ was multiplied by $K_{\lambda_{i}}$, a parameter established by Geraldes et al. [67] to take into consideration additional pressure losses in the feed side due to the SWMM fittings, $PF_{i}$ is the polarization factor, $k_{s_{i}}$ is the solute mass transfer coefficient and $\eta_{i}$ is the dynamic viscosity of solution (8.91 $\times \;10^{-4}$ kg m${}^{-1}$ s${}^{-1}$). The *SEC* was calculated considering the ideal performance (100% efficiency for the electrical engine and high pressure pump), which means that it was calculated with the power input ($P_{{\mathrm{in}}_{i}}$) in the SWMMs.

## 3. Results and Discussion

Table 4 shows the results by SWMM model and position *i* for the operating points that minimize the *SEC*. The SWMMs with lower *A*${}_{0}$ require more $p_{f_{1}}$, $Q_{f_{i}}$ was quite similar for the 9 SWMMs considered, being between 5.7 and 5.9 m${}^{3}$ h${}^{-1}$. In terms of $p_{f_{i}}$, it was observed how the largest pressure drops occur in the SWMMs with lower *h*${}_{i}$ ($7.11\times 10^{-4}$). The $R_{1}$ is lower for the SWMMs with lower *A*${}_{0}$, however the $R_{i}$ drops are lower for the elements with lower *A*${}_{0}$ and so permeate production is more uniform than for the SWMMs with higher *A*${}_{0}$. For all nine SWMMs considered, the $PF_{i}$ decreases with increasing SWMM position. Despite the fact that $v_{{\mathrm{fb}}_{i}}$ decreases along the PV, causing $PF_{i}$ to increase, its effect is outweighed by the reduction of $Q_{p_{i}}$ along the PV in $PF_{i}$, resulting in an overall decrease in *PF*${}_{i}$. In terms of *SEC*, the SWMMs became more energy inefficient as the position increased for the operating points considered. There may be operating points where this behavior changes. The SWMMs with lower $A_{0}$ were observed to have higher *SEC*${}_{i}$ in the first SWMM positions but lower in the last ones. This is due to the more uniform production for the SWMMs with lower $A_{0}$. For SWMMs with higher $A_{0}$, the later positions had lower $Q_{p_{i}}$ implying overall higher *SEC*${}_{i}$.

### 3.1. Implications of $A_{i}$ Reduction on $p_{f_{i}}$

**Figure 2:**
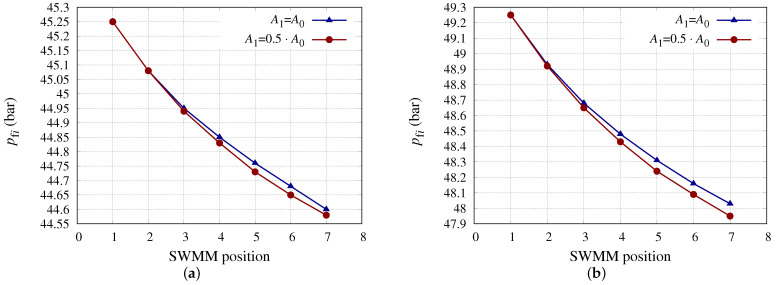
$p_{f_{i}}$ (bar) considering the SWMM in position 1 with different $A_{0}$ reductions. (**a**) $p_{f_{i}}$ (bar) for SWMM model 1 considering the SWMM in position 1 with different $A_{0}$ reductions. (**b**) $p_{f_{i}}$ (bar) for SWMM model 8 considering the SWMM in position 1 with different $A_{0}$ reductions.

Figure 2 shows the $p_{f_{i}}$ for each SWMM position, considering new membranes and a 50% reduction for the coefficient $A_{0}$ at position 1, for SWMM models 1 (Figure 2a) and 8 (Figure 2b). Both models have the same *S*${}_{m}$, but model 1 has a higher $A_{0}$ coefficient than model 8 and a higher *h* (Table 1). This implies that $v_{\mathrm{fb}}$ will be lower for the SWMM in position 1, since this element will produce more permeate and the $Q_{\mathrm{fb}}$ will be lower as well as having a higher *h*, so the pressure drop from one SWMM to the next will be lower, as shown in Figure 2. It should be noted that fouling would not only produce a decrease in *A* but also an increase in the pressure drop from one SWMM to another due to colloid deposition and organic fouling [66]. This effect would be expected to be most pronounced in SWMM 8 since it has a lower *h*. Overall, the pressure drop differences for each of the SWMM models did not show a representative difference between considering new SWMMs and reductions of 50% in the SWMM in first position. For SWMM model 1, the pressure drops along the PV were 0.65 and 0.67 bar considering, respectively, new SWMMs and the SWMM in position 1 with a decrease in $A_{0}$ of 50%, while for SWMM model 8 the corresponding values were 1.22 and 1.30 bar under the same conditions. It should be mentioned that similar expressions were used for λ (Equation (7)). If the membrane manufacturers were to provide these correlations, more accurate estimates could be obtained.

### 3.2. Implications of $A_{i}$ Reduction on *R*${}_{i}$

**Figure 3:**
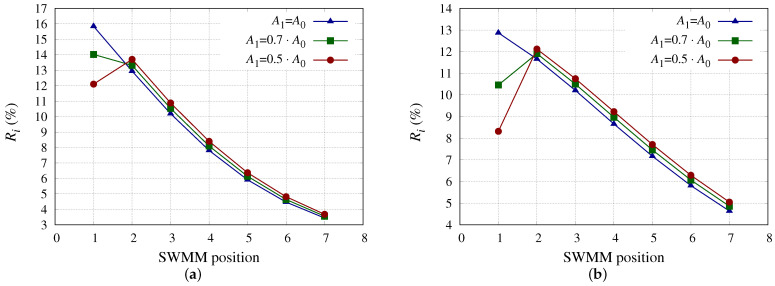
$R_{i}$ (%) considering the SWMM in position 1 with different $A_{0}$ reductions. (**a**) $R_{i}$ (%) for SWMM model 4 considering the SWMM in position 1 with different $A_{0}$ reductions. (**b**) $R_{i}$ (%) for SWMM model 6 considering the SWMM in position 1 with different $A_{0}$ reductions.

Figure 3 shows the $R_{i}$ for each SWMM position, considering new membranes and a 70% and 50% reduction for the coefficient $A_{0}$ at position 1, for SWMM models 4 (Figure 2a) and 6 (Figure 2b). Both models have the same *S*${}_{m}$, *h* but different coefficient $A_{0}$ (Table 1), with this being higher for SWMM model 4 than for model 6. Both models show the same behavior in terms of $R_{i}$; the more $A_{1}$ decreases the more $R_{1}$ decreases and the more $R_{i}$ increases for SWMMs positioned further forward in the PV (positions 2 to 7). This is due to the fact that, for the PV input operating conditions, the lower $\pi_{m_{i}}$ is the higher *R*${}_{i}$ is. This occurs because reducing the $A_{0}$ of the SWMM in the first position, which has the highest *R* (Table 4), causes $Q_{f_{i}}$ to increase for SWMMs in positions 2 to 7.

The decreases in $A_{i}$ have repercussions on the *R* of the PV, and depending on the position of the fouled SWMM will have more or less an impact on $R_{i}$. The coefficient $A_{0}$ also plays an important role in the variations of $R_{i}$ due to the decrease of the aforementioned coefficient. Table 5 and Table 6 show, for SWMM models 2 and 9, respectively, the *R* of the PV, considering different decrements of $A_{0}$ for SWMMs located in different positions. In the operating points considered (Table 2), the SWMM in first position is the most relevant, which is why when the $A_{0}$ decrease affects the SWMM in that position it has a higher impact on the $R_{i}$ of the PV, as can be seen in Table 5 and Table 6. The SWMM models 2 and 9 have the same *S*${}_{m}$ and *h*. However, $A_{0}$ is higher for model 2 than model 9. The $R_{i}$ drop for the $A_{i}$ decrease conditions was higher for the SWMM with lower $A_{0}$, in this case model 9, which means that, under fouling conditions, the RO system with this SWMM will experience greater performance losses than SWMM model 1. However, the impact of fouling in terms of increased *B* coefficient, which would be more favorable for the SWMM model 9, was not evaluated. This could be critical when attention is paid to ions less rejected by SWRO membranes, such as boron and fluoride [69,70,71].

### 3.3. Implications of $A_{i}$ Reduction on *PF*${}_{i}$

**Figure 4:**
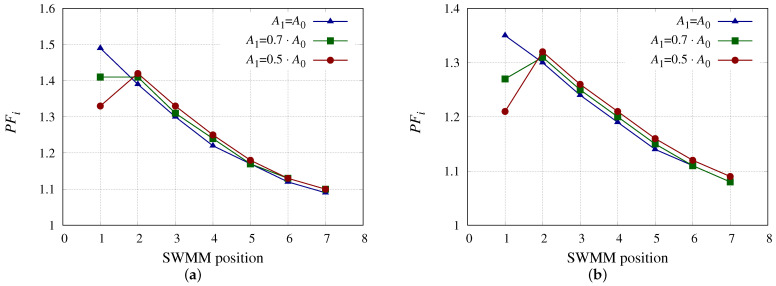
*PF*${}_{i}$ considering the SWMM in position 1 with different $A_{0}$ reductions. (**a**) *PF*${}_{i}$ for SWMM model 3 considering the SWMM in position 1 with different $A_{0}$ reductions. (**b**) *PF*${}_{i}$ for SWMM model 5 considering the SWMM in position 1 with different $A_{0}$ reductions.

**Figure 5:**
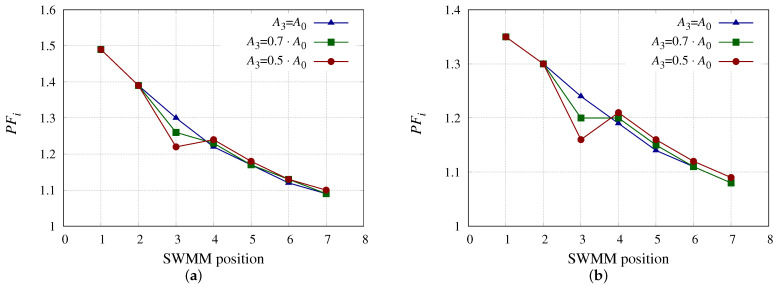
*PF*${}_{i}$ considering the SWMM in position 3 with different $A_{0}$ reductions. (**a**) *PF*${}_{i}$ for SWMM model 3 considering the SWMM in position 3 with different $A_{0}$ reductions. (**b**) *PF*${}_{i}$ for SWMM model 5 considering the SWMM in position 3 with different $A_{0}$ reductions.

Figure 4 show the values of *PF*${}_{i}$ for SWMM models 3 (Figure 4a) and 5 (Figure 4b) considering different decrements of $A_{0}$ for each model. *PF*${}_{i}$ depends on $J_{p}$ and *k* (Equation (15)). The higher $J_{p}$ is the higher *PF*${}_{i}$ is. High values of $J_{p}$ are associated with high permeate production of the SWMM, making the following SWMM receive low $Q_{f_{i}}$ which translates into low $v_{{\mathrm{fb}}_{i}}$ so that Re would also be low as well as *k*, resulting in high values of *PF*${}_{i}$. The SWMM models 3 and 5 have different *S*${}_{m}$, with the value being higher for model 5, while *h* and $A_{0}$ are higher for model 3. *PF*${}_{i}$ values for model 3 were higher than for model 5 due to its higher $A_{0}$ and despite having higher *h*. Because of the decrease in $Q_{p_{1}}$ due to the decrease in $A_{1}$, an increase in *PF*${}_{i}$ was observed in both SWMM models. The increase in *PF*${}_{i}$ in the last SWMM in the PV can bear a significant impact on the $C_{p}$.

Figure 5 shows the impact of the decrease in $A_{3}$ on *PF*${}_{i}$ for the SWMM models studied above. The effects are quite similar to the previous case (Figure 4) with the difference that the SWMM in position 1 has higher values in this case because $A_{1}$ has not decreased. Since $A_{1}$ has not decreased, the SWMM in second position has less $Q_{p}$, and so the *PF*${}_{i}$ is lower than in the previous case for both models (Figure 5a,b). As in the previous case, there is an increase of *PF*${}_{i}$ in the SWMMs positioned after the “fouled” one. It should be noted that the operating points considered, which minimize the *SEC* (Table 2), have quite low $Q_{f_{1}}$ values. This results in a low cross-flow velocity profile and high *PF*${}_{i}$ values. Under fouling conditions (common in RO), it is not advisable to work in operating conditions with high *PF*${}_{i}$ since it promotes the deposition of fouling agents, whether colloidal, biofouling or even scaling, with the latter being common in BWRO where high values of *R* can be reached. Values of $Q_{f_{1}}$ shown in Table 2 are below 6 m${}^{3}$ h${}^{-1}$, while $Q_{f_{1}}$ values above 7 m${}^{3}$ h${}^{-1}$ are normally operated [52,53,55].

### 3.4. Implications of $A_{i}$ Reduction on SEC ${}_{i}$

**Figure 6:**
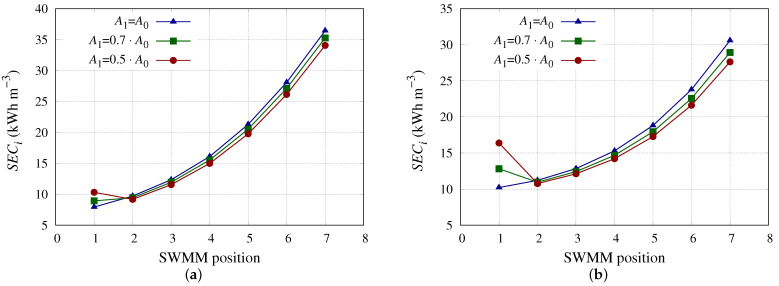
*SEC*${}_{i}$ considering the SWMM in position 1 with different $A_{0}$ reductions. (**a**) *SEC*${}_{i}$ for SWMM model 1 considering the SWMM in position 1 with different $A_{0}$ reductions. (**b**) *SEC*${}_{i}$ for SWMM model 9 considering the SWMM in position 1 with different $A_{0}$ reductions.

The impact on the *SEC* of the PV of the decrements considered for $A_{0}$ is quite low. In fact for SWMM model 1 (highest $A_{0}$ value) and new membranes the *SEC* is 2.665 kWh m${}^{-3}$, and considering $A_{1}$ = 0.5$\;\cdot \;A_{0}$ (which is the most unfavorable case studied) the value is 2.693 kWh m${}^{-3}$. The aforementioned values for the SWMM model 9 (lowest $A_{0}$ value) are 2.853 and 2.913 kWh m${}^{-3}$, respectively. In real RO desalination plants, fouling has a gradual impact on all SWMMs within the PV, causing decreases in $A_{0}$ to occur in them all. This would indeed considerably increase the *SEC* of the desalination plant, as can be seen in previously published studies [52,53,55]. Figure 6 shows the impact of the decrease in $A_{0}$ on *SEC*${}_{i}$. It can be observed that the behaviour is similar for SWMM models 1 and 9. The decrease in $A_{1}$ translated into an increase in *SEC*${}_{1}$, but the following SWMMs experienced a *SEC*${}_{i}$ reduction. However, this reduction was not sufficient to balance the *SEC*${}_{1}$ increase produced in the SWMM in the first position in both SWMM models. The decrease in $A_{1}$ affected the SWMM model more with lower $A_{0}$ (model 9) in terms of *SEC*${}_{i}$. This can be seen in Figure 6a,b, where the points and curves are farther apart for SWMM model 9 (Figure 6b) than for SWMM model 1 (Figure 6a).

**Figure 7:**
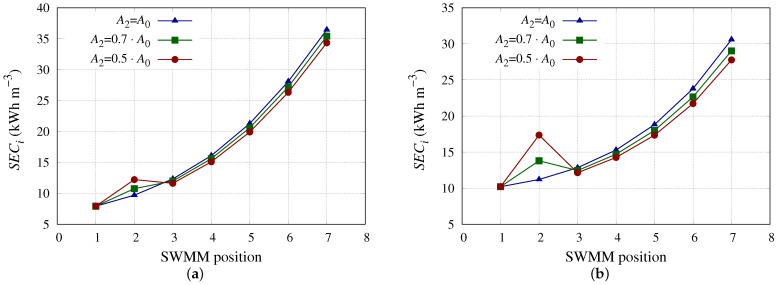
*SEC*${}_{i}$ considering the SWMM in position 2 with different $A_{0}$ reductions. (**a**) *SEC*${}_{i}$ for SWMM model 1 considering the SWMM in position 2 with different $A_{0}$ reductions. (**b**) *SEC*${}_{i}$ for SWMM model 9 considering the SWMM in position 2 with different $A_{0}$ reductions.

Figure 7 shows the impact of decreasing $A_{2}$ in terms of *SEC*${}_{i}$. It shows the same trend as in Figure 6, but causing less impact on the *SEC* of the entire PV. In terms of *SEC*${}_{i}$, in addition to other parameters, the decrease in $A_{1}$ had more impact than this decrease in subsequent SWMMs. In fact, considering decrements of 50% of $A_{i}$ for SWMM model 9, the *SEC* for the entire PV was 2.913 kWh m${}^{-3}$ when the decrement was applied to $A_{1}$, 2.908 kWh m${}^{-3}$ when it was applied to $A_{2}$, 2.904 kWh m${}^{-3}$ when it was applied to $A_{3}$, and so on to a value of 2.891 kWh m${}^{-3}$ when applied to the seventh SWMM. It should be noted that these results are conditioned by accounting for the operating points that minimize *SEC* in which the SWMM in position one is the most relevant. Under different operating conditions where the highest *R* is not found in the SWMM in the first position, different results would be obtained.

## 4. Conclusions

In this study, an evaluation was undertaken concerning how a decrease in the coefficient *A* of an SWMM at different positions within the PV affects the operating point that minimizes the *SEC*. For this purpose, a total of nine SWMM commercial models from three different membrane manufacturers were considered. At this operating point, the decrease in coefficient $A_{1}$ had the greatest impact on the performance of the PV, since this first SWMM produced the most permeate of all PVs for the nine SWMM models studied. When under operating conditions similar to those studied and when the first SWMM is subjected to fouling that produces decreases in the *A* coefficient, this should be the first SWMM to be replaced as it is the most relevant. This does not mean that, regardless of the operating conditions, this is always the case. This will depend on the nominal operating point of the plant. There may be, under other operating conditions, circumstances in which the most relevant SWMM is in a position other than first. As a future line of research, it is proposed to carry out this same study but for wide operating windows and to corroborate the results through experimental studies. In addition, a simultaneous decrease in *A* across various SWMMs should be also considered, as it would closer mimic the operation of real RO desalination plants. This, in turn, would help to determine which SWMMs are more critical allowing a more efficient SWMM replacement in the PV.

## Figures and Tables

**Table 1:** *S*${}_{m}$, *h*, *Rej* (NaCl), *A*${}_{0}$, and *B* of the SWMMs [64].

Number	SWMM Model	*S*_m_ (m${}^{2}$)	*h* (m)	*Rej* (%)	*A*${}_{0}$ (m Pa^−1^ s^−1^)	*B* (m s^−1^)
1	Toray RO TSW-LE-400	37.16	$8.64\times 10^{-4}$	99.69	$8.75\times 10^{-12}$	$2.23\times 10^{-8}$
2	Toray RO TM800V-440	40.88	$7.11\times 10^{-4}$	99.86	$6.00\times 10^{-12}$	$1.45\times 10^{-8}$
3	Toray RO TM800V-400	37.16	$8.64\times 10^{-4}$	99.86	$6.08\times 10^{-12}$	$1.46\times 10^{-8}$
4	Hydraunautics SWC6-LD-400	37.16	$8.64\times 10^{-4}$	99.69	$8.44\times 10^{-12}$	$2.20\times 10^{-8}$
5	Hydraunautics SWC4-MAX	40.88	$7.11\times 10^{-4}$	99.85	$3.64\times 10^{-12}$	$1.17\times 10^{-8}$
6	Hydraunautics SWC4-LD	37.16	$8.64\times 10^{-4}$	99.85	$3.58\times 10^{-12}$	$1.17\times 10^{-8}$
7	Filmtec^™^ SW30XLE-400	37.16	$7.11\times 10^{-4}$	99.86	$6.06\times 10^{-12}$	$1.45\times 10^{-8}$
8	Filmtec^™^ SW30HRLE-400	37.16	$7.11\times 10^{-4}$	99.85	$3.75\times 10^{-12}$	$1.19\times 10^{-8}$
9	Filmtec^™^ SW30XHR-440	40.88	$7.11\times 10^{-4}$	99.86	$3.19\times 10^{-12}$	$9.86\times 10^{-9}$

**Table 2:** $p_{f}$, $Q_{f}$, *R*, $C_{p}$ and $p_{b}$ to obtain minimum *SEC* with $C_{f}$ = 30 g L${}^{-1}$ and considering 7 SWMMs in series [64].

	SWMM Model								
**Parameter**	**1**	**2**	**3**	**4**	**5**	**6**	**7**	**8**	**9**
$p_{f}$ (bar)	45.25	45.25	48.50	45.5	49.50	49.50	46.75	49.25	49.50
$Q_{f}$ (m${}^{3}$ h${}^{-1}$)	5.7	5.7	5.9	5.7	5.9	5.7	5.8	5.8	5.8
*R* (%)	47.16	46.96	49.12	47.35	49.1	47.36	48.06	48.21	48.2
$C_{p}$ (mg L${}^{-1}$)	378.82	263.88	238.22	373.43	201.75	195.62	235.14	190.08	172.54
$p_{b}$ (bar)	44.54	44.17	47.76	44.79	48.35	48.75	45.45	47.91	48.35
*SEC* (kWh m${}^{-3}$)	2.665	2.677	2.743	2.670	2.801	2.903	2.702	2.838	2.853

**Table 3:** Equations based on solution–diffusion transport phenomena in the SWRO process [64].

Permeate flow	(1) $Q_{p}=A\;\cdot \;TMP\;\cdot \;S_{m}$
Water permeability coefficient	(2) $A_{i}=A_{0}\;\cdot \;TCF\;\cdot \;FF$
Temperature correction factor (If $T⩾25{\;}^{\circ }$C)	(3) $$TCF=\exp\left[2640\;\cdot \;\left(\frac{1}{298}-\frac{1}{273+T}\right)\right]$$
Temperature correction factor (If $T⩽25{\;}^{\circ }$C)	(4) $$TCF=\exp\left[3020\;\cdot \;\left(\frac{1}{298}-\frac{1}{273+T}\right)\right]$$
Transmembrane pressure	(5) $\mathrm{TMP}=\left(\Delta p-\Delta \pi \right)=p_{f}-\frac{\Delta p_{\mathrm{fb}}}{2}-p_{p}-\pi_{m}+\pi_{p}$
Feed-brine pressure drop	(6) $\Delta p_{\mathrm{fb}}=\lambda \;\cdot \;L\;\cdot \;\frac{\rho_{\mathrm{fb}}}{d_{h}}\frac{v_{{\mathrm{fb}}^{2}}}{2}$
Friction factor [67,68]	(7) $\lambda =K_{\lambda }\;\cdot \;6.23{Re}^{-0.3}$
Reynolds number	(8) $Re=\frac{\rho_{\mathrm{fb}}\;\cdot \;\nu_{\mathrm{fb}}\;\cdot \;d_{h}}{\eta }$
Hydraulic diameter	(9) $d_{h}=\frac{4\varepsilon }{\frac{2}{h}+\left(1-\varepsilon \right)\frac{8}{h}}$
Feed-brine solution density [33]	(10) $\rho_{\mathrm{fb}}=498.4\;\cdot \;M+\sqrt{248,400+752.4\;\cdot \;C_{\mathrm{fb}}\;\cdot \;M}$
Empirical parameter [33]	(11) $M=1.0069-2.757\times 10^{-4}\;\cdot \;T_{\mathrm{fb}}$
Feed-brine concentration	(12) $C_{\mathrm{fb}}=C_{f}\;\cdot \;\left(\frac{1+\frac{C_{b}}{C_{f}}}{2}\right)$
Osmotic pressure	(13) $\pi =4.54047\;\cdot \;{\left(10^{3}\;\cdot \;C/\left(M_{s}\;\cdot \;\rho \right)\right)}^{0.987}$
Concentration on membrane surface	(14) $C_{m}=C_{\mathrm{fb}}\;\cdot \;PF$
Polarization factor	(15) ${PF}_{i}=\frac{C_{m}}{C_{\mathrm{fb}}}=e^{\frac{J_{p}}{k}}$
Sherwood number [68]	(16) $\mathrm{Sh}=0.065\;\cdot \;{Re}^{0.875}\;\cdot \;{Sc}^{0.25}=\frac{k\;\cdot \;d_{h}}{D_{s}}$
Schmidt number	(17) $Sc=\frac{\eta }{\rho_{\mathrm{fb}}\cdot D_{s}}$
Solute diffusivity	(18) $D_{s}=\left(0.72598+0.023087T_{\mathrm{fb}}+0.00027657T_{\mathrm{fb}}^{2}\right)\times 10^{-9}$
Permeate concentration	(19) $C_{p}=B\;\cdot \;PF\;\cdot \;TCF\;\cdot \;\frac{S_{m}}{Q_{p}}\;\cdot \;\left(\frac{C_{f}\;\cdot \;\left(1+CF\right)}{2}\right)$
Concentration factor	(20) $CF=\frac{100-R\;\cdot \;(1-\mathrm{Rej})}{100-R}$
Flux recovery	(21) $R=100\;\cdot \;\frac{Q_{p}}{Q_{f}}$
Specific energy consumption	(22) $SEC_{i}=\frac{P_{{\mathrm{in}}_{i}}}{Q_{p_{i}}}$
Power input	(23) $P_{{\mathrm{in}}_{i}}=p_{f_{i}}Q_{f_{i}}\gamma_{f_{i}}$

**Table 4:** $p_{f_{i}}$, $Q_{f_{i}}$, $R_{i}$, *PF*${}_{i}$ and *SEC*${}_{i}$ per SWMM model and position considering non-fouled SWMMs.

SWMM Model	Parameter	SWMM Position						
		1	2	3	4	5	6	7
1	$p_{f_{i}}$ (bar)	45.25	45.08	44.95	44.85	44.76	44.68	44.60
	$Q_{f_{i}}$ (m${}^{3}$ h${}^{-1}$)	5.70	4.80	4.18	3.75	3.46	3.26	3.12
	$R_{i}$ (%)	15.86	12.90	10.12	7.74	5.84	4.42	3.40
	*PF* ${}_{i}$	1.51	1.38	1.28	1.20	1.14	1.11	1.08
	*SEC*${}_{i}$ (kWh m${}^{-3}$)	7.93	9.71	12.34	16.10	21.29	28.08	36.44
2	$p_{f_{i}}$ (bar)	45.25	44.99	44.80	44.64	44.50	44.38	44.27
	$Q_{f_{i}}$ (m${}^{3}$ h${}^{-1}$)	5.70	4.80	4.17	3.74	3.45	3.26	3.12
	$R_{i}$ (%)	15.86	13.07	10.25	7.73	5.70	4.17	3.09
	*PF* ${}_{i}$	1.40	1.30	1.22	1.16	1.11	1.08	1.06
	*SEC*${}_{i}$ (kWh m${}^{-3}$)	7.93	9.56	12.14	16.04	21.69	29.56	39.80
3	$p_{f_{i}}$ (bar)	48.50	48.32	48.18	48.07	47.98	47.90	47.82
	$Q_{f_{i}}$ (m${}^{3}$ h${}^{-1}$)	5.90	4.98	4.32	3.86	3.52	3.29	3.12
	$R_{i}$ (%)	15.52	13.23	10.84	8.59	6.65	5.09	3.88
	*PF* ${}_{i}$	1.49	1.39	1.30	1.22	1.17	1.12	1.09
	*SEC*${}_{i}$ (kWh m${}^{-3}$)	8.68	10.15	12.35	15.54	20.04	26.14	34.24
4	$p_{f_{i}}$ (bar)	45.50	45.33	45.20	45.10	45.00	44.93	44.86
	$Q_{f_{i}}$ (m${}^{3}$ h${}^{-1}$)	5.70	4.80	4.18	3.75	3.46	3.25	3.11
	$R_{i}$ (%)	15.84	12.94	10.19	7.81	5.91	4.47	3.44
	*PF* ${}_{i}$	1.50	1.38	1.28	1.20	1.15	1.11	1.08
	*SEC*${}_{i}$ (kWh m${}^{-3}$)	7.98	9.73	12.32	16.04	21.15	27.92	36.22
5	$p_{f_{i}}$ (bar)	49.50	49.22	49.01	48.84	48.69	48.56	48.45
	$Q_{f_{i}}$ (m${}^{3}$ h${}^{-1}$)	5.90	5.05	4.40	3.92	3.57	3.32	3.13
	$R_{i}$ (%)	14.37	12.82	10.94	8.96	7.11	5.50	4.20
	*PF* ${}_{i}$	1.35	1.30	1.24	1.19	1.14	1.11	1.08
	*SEC*${}_{i}$ (kWh m${}^{-3}$)	9.57	10.66	12.44	15.14	19.02	24.53	32.04
6	$p_{f_{i}}$ (bar)	49.50	49.33	49.19	49.07	48.98	48.89	48.82
	$Q_{f_{i}}$ (m${}^{3}$ h${}^{-1}$)	5.70	4.97	4.39	3.94	3.60	3.34	3.15
	$R_{i}$ (%)	12.87	11.66	10.21	8.66	7.17	5.81	4.64
	*PF* ${}_{i}$	1.39	1.34	1.28	1.23	1.18	1.14	1.11
	*SEC*${}_{i}$ (kWh m${}^{-3}$)	10.68	11.75	13.38	15.74	18.98	23.37	29.23
7	$p_{f_{i}}$ (bar)	46.75	46.44	46.20	46.01	45.85	45.71	45.58
	$Q_{f_{i}}$ (m${}^{3}$ h${}^{-1}$)	5.80	4.87	4.22	3.77	3.47	3.26	3.11
	$R_{i}$ (%)	15.98	13.36	10.62	8.11	6.03	4.42	3.26
	*PF* ${}_{i}$	1.41	1.32	1.24	1.17	1.12	1.09	1.06
	*SEC*${}_{i}$ (kWh m${}^{-3}$)	8.13	9.66	12.08	15.76	21.12	28.73	38.84
8	$p_{f_{i}}$ (bar)	49.25	48.93	48.68	48.48	48.31	48.16	48.03
	$Q_{f_{i}}$ (m${}^{3}$ h${}^{-1}$)	5.80	5.00	4.38	3.91	3.57	3.32	3.14
	$R_{i}$ (%)	13.80	12.38	10.64	8.80	7.04	5.49	4.23
	*PF* ${}_{i}$	1.34	1.29	1.24	1.19	1.14	1.11	1.08
	*SEC*${}_{i}$ (kWh m${}^{-3}$)	9.91	10.98	12.71	15.30	19.06	24.37	31.54
9	$p_{f_{i}}$ (bar)	49.50	49.23	49.02	48.84	48.70	48.57	48.45
	$Q_{f_{i}}$ (m${}^{3}$ h${}^{-1}$)	5.80	5.02	4.41	3.94	3.59	3.33	3.14
	$R_{i}$ (%)	13.47	12.20	10.61	8.87	7.19	5.67	4.40
	*PF* ${}_{i}$	1.32	1.28	1.23	1.19	1.15	1.11	1.08
	*SEC*${}_{i}$ (kWh m${}^{-3}$)	10.21	11.21	12.83	15.30	18.81	23.79	30.59

**Table 5:** *R* (%) of the PV (7 SWMMs in series) with SWMM model 2 (Table 1) considering fouled SWMMs located in different positions.

Position of the Fouled SWMM	*R* (%) for SWMM Model 2				
	$A_{i}$ = 0.9$A_{0}$	$A_{i}$ = 0.8$A_{0}$	$A_{i}$ = 0.7$A_{0}$	$A_{i}$ = 0.6$A_{0}$	$A_{i}$ = 0.5$A_{0}$
1	46.87	46.77	46.65	46.51	46.34
2	46.88	46.80	46.69	46.57	46.43
3	46.89	46.82	46.73	46.62	46.49
4	46.90	46.83	46.75	46.65	46.54
5	46.91	46.84	46.77	46.68	46.58
6	46.91	46.86	46.79	46.72	46.62
7	46.92	46.87	46.82	46.75	46.67

**Table 6:** *R* (%) of the PV (7 SWMMs in series) with SWMM model 9 (Table 1) considering fouled SWMMs located in different positions.

Position of the Fouled SWMM	*R* (%) for SWMM Model 2				
	$A_{i}$ = 0.9$A_{0}$	$A_{i}$ = 0.8$A_{0}$	$A_{i}$ = 0.7$A_{0}$	$A_{i}$ = 0.6$A_{0}$	$A_{i}$ = 0.5$A_{0}$
1	48.03	47.86	47.66	47.44	47.19
2	48.05	47.89	47.71	47.51	47.28
3	48.07	47.92	47.76	47.57	47.35
4	48.08	47.95	47.79	47.62	47.42
5	48.09	47.96	47.82	47.66	47.47
6	48.10	47.98	47.85	47.69	47.51
7	48.10	47.99	47.87	47.73	47.56

## Data Availability

Not applicable.

## Abbreviations

The following abbreviations are used in this manuscript:

Nomenclature	
*A*	Water permeability coefficient (m Pa${}^{-1}$ s${}^{-1}$)
*B*	Ion permeability coefficient (m s)
*C*	Concentration (g l^−1^)
CF	Concentration factor
*D*	Diffusivity (m^2^ s^−1^)
$d_{h}$	Hydraulic diameter (m)
FF	Flow factor
*h*	Feed spacer height
*J*	Flow per unit area (m^3^ m^−2^ d^−1^)
$K_{\lambda }$	Additional pressure losses factor
*k*	Mass transfer coefficient
*L*	Length of the spiral wound membrane module (m)
*m*	Molal concentration (mol kg^−1^)
$P_{\mathrm{in}}$	Power input (kW)
PF	Polarization factor
PV	Pressure vessel
*p*	Pressure (Pa)
*Q*	Flow (m^3^ s^−1^)
*R*	Flow recovery (%)
Re	Reynolds number
Rej	Rejection (%)
RO	Reverse osmosis
*S*${}_{m}$	Membrane surface (m^2^)
*Sc*	Schmidt number
*SEC*	Specific energy consumption (kW h m^−3^)
*Sh*	Sherwood number
SWMM	Spiral wound membrane module
*T*	Temperature (${}^{\circ }$C)
*TCF*	Temperature correction factor
*TMP*	Transmembrane pressure (Pa)
Greek letters	
ε	Porosity of the cross-sectional area in the feed channel
η	Dynamic viscosity (kg m^−1^ s)
γ	Specific weight (N m${}^{-3}$)
λ	Friction factor
ν	Velocity (m s^−1^)
π	Osmotic pressure (Pa)
ρ	Density ($kg\;m^{-3}$)
Δp	Pressure gradient (Pa)
Δπ	Osmotic pressure gradient (Pa)
Subscripts	
0	Initial
av	Average
f	Feed
fb	Feed-brine
*i*	Position of the SWMM within the PV
m	Membrane
p	Permeate
b	Brine
s	Solute
